# Low-dose heparin as treatment for early disseminated intravascular coagulation during sepsis: A prospective clinical study

**DOI:** 10.3892/etm.2013.1466

**Published:** 2013-12-31

**Authors:** XIAO-LI LIU, XIAO-ZHI WANG, XIU-XIANG LIU, DONG HAO, YASAMAN JALADAT, FENG LU, TING SUN, CHANG-JUN LV

**Affiliations:** 1 Department of Respiratory Disease, Hospital of Binzhou Medical University, Binzhou, Shandong 256603, P.R. China; 2 Department of Otolaryngology-HNS, Case Western Reserve University, Cleveland, OH 44106, USA

**Keywords:** sepsis, low-dose heparin, coagulation, treatment, clinical trial, disseminated intravascular coagulation

## Abstract

The present study aimed to investigate whether low-dose heparin improves the condition of patients suffering from early disseminated intravascular coagulation (pre-DIC) during sepsis. In total, 37 patients were randomly divided into low-dose heparin intervention and control groups. The heparin group received a low-dose of heparin for 5–7 days, while the other group received only saline. The two groups were treated for sepsis. Blood samples were collected at various times and acute physiology and chronic health evaluation (APACHE)-II scores were recorded at day 1 and 7. In addition, the number of days applying mechanical ventilation and in the intensive care unit (ICU) were recorded, as well as the 28-day mortality rate. APACHE-II scores in the two groups decreased following treatment, however, scores in the heparin group decreased more significantly. Prothrombin fragment and thrombin-antithrombin complex levels in the heparin group were significantly decreased. In addition, the number of days applying a ventilator was fewer and the total stay in ICU was significantly shorter compared with the control group. Significantly fewer complications were observed in the heparin group, however, there was no significant difference in the 28-day mortality rate. In conclusion, low-dose heparin improves the hypercoagulable state of sepsis, which subsequently reduces the incidence of DIC or multiple organ dysfunction syndrome, decreasing the number of days of mechanical ventilation and hospitalization.

## Introduction

Systemic inflammatory responses, coagulation/fibrinolytic system disorders and immune dysfunction are among the pathophysiological features common during sepsis (1). A previous study (1) has shown that the severity and prognosis of sepsis are closely associated with blood coagulation disorders or disseminated intravascular coagulation (DIC). Multiple organ dysfunction syndrome (MODS), which is a severe consequence of DIC, results in high mortality of septic patients. Early manifestation of DIC is not well diagnosed. The hypercoagulable state of early DIC has recently been named pre-DIC, characterized by a predisposing factor for DIC and fibrinolytic coagulation abnormalities, but with insufficient criteria for diagnosing DIC. Successful diagnosis of pre-DIC may prevent DIC, as underlying diseases and hematological abnormalities may be managed.

Three large-scale multi-center clinical trials were performed to investigate the efficacy of coagulation inhibitors as treatments for sepsis. In phase III clinical trials, recombinant human activated protein C (APC) was found to significantly reduce the 28-day mortality rate (2,3). Application of APC was strongly recommended by the Society of Critical Care Medicine (SCCM) for the treatment of severe septic patients, who had an acute physiology and chronic health evaluation (APACHE)-II score of >25 points (4). However, the cost of this treatment is >6,000 US dollars for each course, which makes it extremely expensive to apply for the majority of patients. Heparin, as an anticoagulant, which, not only inhibits the activation of the coagulation system, but is also an anti-inflammatory and immunomodulatory agent, has been widely used during DIC treatment and in the prevention and treatment of thrombotic diseases. It is easy to obtain and inexpensive. Several non-randomized clinical studies indicated that heparin partially inhibits sepsis by activating the coagulation system (5). However, there is a lack of randomized controlled prospective clinical studies. Therefore, the aim of present study was to investigate the effects of low-dose heparin treatment in patients with sepsis and pre-DIC to determine whether this treatment improves the coagulation status, MODS and/or the prognosis of patients.

## Materials and methods

### Reagents and patient selection

Patients were selected according to the diagnostic criteria for sepsis of the American Thoracic Society, presented in 1992 (American College of Chest Physicians/SCCM) (6) and the pre-DIC diagnostic criteria established at the Seventh Country Thrombosis and Bleeding Meeting in China in 1999. Informed consent was obtained from the patients and the study was approved by the local Ethics Review Committee of the Hospital of Binzhou Medical University (Binzhou, China).

Exclusion criteria included pregnancy/breast-feeding, age <16 or >70 years, requiring or receiving anticoagulant therapy for thrombotic disease, requiring blood or peritoneal dialysis for chronic renal failure, severe chronic lung*,* kidney or liver diseases, contraindications for the use of heparin (congenital bleeding disorders), serious head injury, intracranial surgery or stroke <28 days prior to enrollment, brain arteriovenous malformation, cerebral aneurysm or a history of extensive damage of the central nervous system. Following selection, patients were randomly divided into the heparin and control treatment groups.

F1+2 (prothrombin fragment 1+2), TAT (thrombin-antithrombin complex), AT-III (antithrombin-III) and PAI-1 (plasminogen activator inhibitor-1) levels were determined using double-antibody sandwich ELISA kits (Age Diagnostics Laboratories Ltd., Boca Raton, FL, USA), according to the manufacturer’s instructions.

### Study design and procedure

The study was performed as a randomized, double-blind, placebo controlled single-center clinical trial. Following selection, patients were randomly divided into the heparin or control treatment groups.

In the heparin group, 70 U/kg/24 h heparin was administered by continuous infusion for 5–7 days. The input rate and dose were adjusted according to the activated partial thromboplastin time (APTT), which extended to 2 or 3 times. In the placebo group, an equal amount of saline was administered. The two groups were treated according to the sepsis cluster treatment guidelines (4).

At 12, 18, 24, 48 and 72 h and at day 7 following treatment, serum samples were collected and anticoagulated using sodium citrate. Plasma F1+2, TAT, AT-III and PAI-1 levels were determined as the primary endpoint measures using sandwich ELISA assays. Platelet count (PLT), prothrombin time (PT), APTT, fibrinogen (Fib) and other indicators were investigated in the hospital laboratory. At the same time, the second endpoint measurements, including APACHE-II-scores, were recorded and calculated for each patient. In addition, the number of days receiving mechanical ventilation and undergoing treatment in the intensive care unit (ICU) were recorded, as well as the 28-day mortality rate for the two groups. Moreover, the number of patients suffering from MODS or DIC was established.

### Statistical analysis

Data were analyzed using SPSS 11.5 (SPSS, Inc., Chicago, IL, USA) and are expressed as mean ± SD. Coagulation indicators of the two groups were compared using multi-factor repeated measurement analysis of variance. P<0.05 was considered to indicate a statistically significant difference.

## Results

### Patients

**Figure 1:**
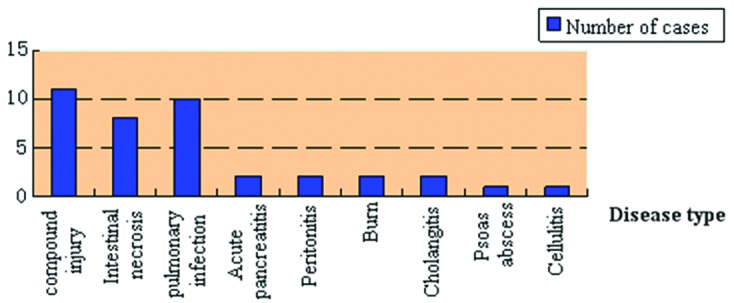
Hospitalization causes of the patients included in the study.

In total 37 sepsis patients (21 males, 16 females; average age, 49.3 years), treated between June 2008 and March 2009 in the ICU at Binzhou Hospital, were enrolled in the study. A list of the underlying diseases of the patients is summarized in Fig. 1. Patients were randomly assigned to a heparin (n=22) or control (n=15) group. There were no clinically significant differences in the APACHE-II scores between the two groups (Table I). However, after seven days, the APACHE-II scores had significantly decreased in the two groups with the decrease in the heparin group being significantly higher (P=0.044; Table II).

**Figure 2:**
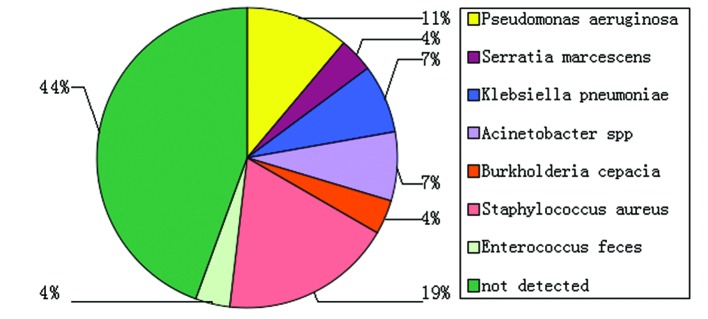
Various pathogens identified in the sputum or body fluids of the patients.

Sputum or body fluids from 15 patients of each group were cultured and found positive for the following micro-organisms: *Pseudomonas aeruginosa, Viscous marcescens, Klebsiella pneumoniae, Powell acinetobacter, Burkholderia cepacia, Enterococcus feces* and *Staphylococcus aureus* (Fig. 2).

### Indicators of coagulation activation

PT, APTT, Fib concentration and PLT were assessed prior to and following treatment. These parameters were not found to be significantly different in the two groups. However, the F1+2 level increased significantly in the heparin group during the first 18 h, as compared to the baseline (0 h) measurement and then decreased significantly at 72 h following the start of treatment. TAT levels increased at 48 h and then also decreased significantly at day 7. AT-III and PAI-1 levels in the groups did not change significantly during treatment (P>0.05; Tables IV and V).

### Mechanical ventilation time, number of days in the ICU and 28-day mortality

Patients receiving low-dose heparin treatment required significantly less mechanical ventilation and fewer days in the ICU, as compared to the control group (P=0.017 and 0.048, respectively). After 28 days, 13 patients had succumbed to the disease, of whom 7 (31.8%) were in the heparin group and 6 (40%) in the control group (Table II).

### Occurrence of complications

Significant differences were observed for the incidence of MODS and DIC between the two groups (P<0.05; Table II).

## Discussion

Results of an epidemiological study in the USA found sepsis to have a high incidence of ~0.3%, which is on the increase at a rate of 1.5% annually (7). Financial issues are involved in the treatment of sepsis, with the annual cost being 17.4 billion US dollars. In addition, the mortality rate of sepsis is high, with findings of a previous study indicating that despite intensive care, mortality remains at 30–50% (8). More than half of the patients with sepsis present with coagulation factor abnormalities and the incidence of DIC is >20%. In cases of DIC incidence, the mortality rate is almost 63% (9). It has been confirmed that DIC leads to ischemic injury of vital organs (10) and eventually to MODS (11). Therefore, treating the coagulopathy state is necessary to improve multiple organ function, which may also be a promising approach for treating sepsis.

Sepsis involves the production of endotoxin by Gram-positive bacteria, a key mediator of the inflammatory response during septic shock. In the current study, 56% of the cases detected pathogens, 23% of which were gram-positive bacteria (*Staphylococcus aureus* being the most common).

Coagulation activation is an important feature in the pathogenesis of sepsis (9). Thrombin generation is a key step in an activated coagulation system. Due to the extremely short half-life of thrombin, it is difficult to determine activated thrombin levels in blood. Therefore, other molecular markers were used to confirm thrombin generation. In the present study, four molecular markers were selected to detect the early stages of coagulation. The markers are produced at various stages and appear earlier than PT, APTT and platelet abnormalities. In addition, the markers were selected to help diagnose the emergence of pre-DIC. These early indicators included F1+2, TAT, fibrinopeptide A and soluble fibrin monomer complex. F1+2 directly reflects the amount of generated thrombin, while the remaining three markers only partially reflect thrombin generation. Therefore, F1+2 is considered the most sensitive indicator of thrombin generation (12).

Heparin is a sulfated polysaccharide with a heterogeneous structure and complex polymerization (MW, 3–57 kDa). Heparin binds to AT-III, causing a conformational change that increases the flexibility of the reactive site loop, activating AT-III. The activated AT-III then inactivates thrombin and other proteases, including factor Xa. Heparin also binds platelets to induce platelet aggregation, resulting in a strong anticoagulant effect (13). In the present study, the subjects were in an early stage of DIC, during which blood is hypercoagulable, platelets are activated and activation of coagulation has already been initiated. In addition, during the pre-DIC period, there is no extensive micro-thrombosis, fibrinolysis, consumption or degradation of platelets and clotting factors. During low-dose heparin therapy, F1+2 showed a gradual increase, followed by a decrease over the first 24 h. At 72 h and 7 days, it was found to be present in significantly lower amounts (P<0.05). Similarly, TAT was initially increased, but subsequently decreased and eventually was reduced to only half the initial level at day 7, following initiation of low-dose heparin therapy (P<0.05). This was consistent with the observation that therapy led to the activation of coagulation (12). Heparin is not only involved with the coagulation factor Xa, but also collaborates with AT to inhibit thrombin. The heparin dose used to inhibit factor Xa was much lower than that required to inactivate thrombin, thus it is likely that the effectiveness of low-dose heparin is dependent on this mechanism to prevent thrombosis. Formation of thrombin is reduced by inhibiting factor Xa and F1+2, which explains the decrease in thrombin levels following a reduction in factor Xa. As a result, TAT decreases, which is consistent with the observations in the present study of decreased F1+2 and TAT levels.

AT-III is a member of the serine protease inhibitor family, which is the most important plasma inhibitor of the coagulation system. It is capable of inhibiting all serine proteases at a low speed, particularly factor Xa and thrombin. In the presence of heparin, inhibition speed increases significantly. AT is also known as a heparin cofactor. However, activity of AT is affected by the pH of the body, which means that loss of the inhibitor increases during acidosis. If acidosis is enhanced, AT activity significantly improves (14). During sepsis, elastase released from activated neutrophils inactivates AT. Clearance of TAT complexes, a process that occurs rapidly, results in the clearance of AT. Heparin may significantly increase AT activity. In the current study, following the administration of low doses of heparin to the patients, AT levels increased gradually. However, the patients were undergoing sepsis, which is usually associated with disturbances in oxygen supply and demand, an acid-base imbalance and high blood lactate (15). AT-III is therefore in a low state of activity. Moreover, with patients in a hypercoagulable state, when AT consumption and degradation are increased, AT-III recovery and improvement of oxygen metabolism are expected. This may be the reason for the recovery of AT-III not being observed following treatment.

PAI-1 is mainly synthesized by endothelial cells and secreted in the subendothelial space. It is the most important factor of a variety of PAIs and functions as an inhibitor of tissue plasminogen activator and urokinase. In the present study, PAI-1 levels of heparin-treated patients did not decrease significantly following heparin administration. This finding indicates that the concentration of plasma PAI-1 is important in the maintenance of patients with sepsis and DIC (16).

The current study has demonstrated that APACHE-II scores of the two treatment groups significantly decreased, while low-dose heparin intervention improved the scores more significantly. Low-dose heparin shortened the normal coagulation time by preventing clotting. Thus, heparin treatment not only improved blood circulation and oxygen supply, but also lowered the necessity of mechanical ventilation significantly (P=0.048). Altogether, this resulted in a shorter duration of stay at the hospital. Improvements in microcirculation not only blocked the development of DIC, but also decreased MODS.

A limitation of this study is the small number of patients enrolled in the present study. In addition, a few patients exited the study prematurely, not allowing time for follow-up. However, the study was conducted as a prospective clinical trial, which is uncommon and allows for several conclusions. No significant differences were observed between the two treatment groups, which confirms the results of an international clinical study aiming to determine the effects of low-dose heparin treatment during sepsis (17,18). The clinical results obtained indicate that shortening the stay in the ICU and decreasing the need for mechanical ventilation reduces the treatment costs of sepsis patients. In addition, the application of low-dose heparin is relatively inexpensive and safe, indicating the suitability for the early treatment of sepsis.

## Figures and Tables

**Table I:** Patient age, gender and APACHE-II scores in the two treatment groups.

Characteristics	Heparin (n=22)	Control (n=15)	P-value
Male:female	12:10	9:6	0.254
Age, years	48.86±14.30	47.47±14.68	0.576
APACHE-II score	20.82±6.50	21.0±6.69	0.935

APACHE, acute physiology and chronic health evaluation score.

**Table II:** Clinical indicators between the low-dose heparin treatment and control groups.

Clinical indicators	Control (n=15)	Heparin (n=22)	P-value
APACHE-II score			
0 h	21.00±6.69	20.82±6.50	0.935
7 days	18.26±6.12	15.68±6.16	
0 h-7 days	2.73±2.49	5.13±4.48	0.044
Days in ICU	14.20±7.31	9.00±5.35	0.017
Days of applying ventilator	11.73±8.34	7.00±5.74	0.048
28-day mortality (%)	6/15 (40)	7/22 (31.8)	0.434
Complications morbidity, n (%)			
MODS	11 (73.3)	8 (36.4)	0.030
DIC	6 (40.0)	2 (9.1)	0.034

Results are expressed as means ± SD. APACHE, acute physiology and chronic health evaluation score; ICU, intensive care unit; MODS, multiple organ dysfunction syndrome; DIC, disseminated intravascular coagulation.

**Table III:** Coagulation parameters prior to and following low-dose heparin treatment.

Group	Time	PT, sec	APTT, sec	Fib, g/l	PLT, ×10^9^/l
Heparin	0 h	13.38±2.83	27.89±8.62	4.42±1.61	163.18±84.60
	7 days	13.41±1.81	40.03±14.21	3.88±0.94	158.27±93.21
Control	0 h	13.44±2.51	26.71±10.88	4.04±1.95	169.67±59.68
	7 days	13.49±2.52	39.41±13.79	4.65±1.87	185.27±84.78

PT, prothrombin time; APTT, activated partial thromboplastin time; Fib, fibrinogen; PLT, platelet count.

**Table IV:** Activated coagulation parameters under low-dose heparin treatment.

	F1+2, nmol/l		TAT, ng/ml	
		
Time	Heparin	Control	Heparin	Control
0 h	1.66±0.69	1.68±0.81	4.21±2.15	4.38±2.19
12 h	1.65±0.61	1.57±0.60	4.58±3.05	4.12±1.87
18 h	1.82±0.77*	1.58±0.62	4.82±2.89	4.24±1.63
24 h	1.94±0.99	1.57±0.51	4.05±3.67	4.27±1.39
48 h	1.84±1.14	1.59±0.70	4.78±4.48*	4.34±1.43
72 h	1.22±0.61*	1.61±0.65	3.30±2.17	4.28±1.31
7 days	0.88±0.48*	1.28±0.56*	2.44±1.51*	3.91±1.49

* P<0.05, vs. 0 h measurement.

F, prothrombin fragment; TAT, thrombin-antithrombin complex.

**Table V:** Anti-coagulation and fibrinolysis parameters under low-dose heparin treatment.

	AT-III, IU/ml		PAI-1, ng/ml	
		
Time	Heparin	Control	Heparin	Control
0 h	3.92±2.07	3.94±1.44	25.76±8.58	27.09±7.84
12 h	3.73±1.76	3.86±1.28	26.02±11.06	25.46±6.80
18 h	3.82±1.96	3.84±1.46	25.02±8.71	26.31±7.68
24 h	3.82±1.45	3.83±1.54	25.35±9.08	23.73±6.83
48 h	4.13±1.45	4.04±1.53	26.44±9.12	23.48±4.99
72 h	4.43±1.38	4.13±1.47	22.64±10.16	23.77±6.46
7 days	4.48±1.52	4.17±1.21	20.74±8.13	23.45±7.29

AT-III, antithrombin-III; PAI-1, plasminogen activator inhibitor-1.
